# Adverse events following immunization with pentavalent vaccine: experiences of newly introduced vaccine in Iran

**DOI:** 10.1186/s12865-017-0226-8

**Published:** 2017-08-23

**Authors:** Manoochehr Karami, Pegah Ameri, Jalal Bathaei, Zeinab Berangi, Tahereh Pashaei, Ali Zahiri, Seyed Mohsen Zahraei, Hussein Erfani, Koen Ponnet

**Affiliations:** 1 Social Determinants of Health Research Center, Hamadan University of Medical Sciences, Hamadan, Iran; 2 Department of Epidemiology, School of Public Health, Hamadan University of Medical Sciences, Hamadan, Iran; 3 Deputy for Health, Hamadan University of Medical Sciences, Hamadan, Iran; 4 Social Determinants of Health Research Center, Kurdistan University of Medical Sciences, Sanandaj, Iran; 5 Center for Communicable Diseases Control, Ministry of Health, Tehran, Iran; 6 Department of Communication Studies, Ghent University, Ghent, Belgium; 7 Faculty of Social Sciences, University of Antwerp, Antwerp, Belgium

**Keywords:** Immunization, Vaccine, Adverse reactions, Iran

## Abstract

**Background:**

The most important factors that affect the incidence of vaccine-related complications are the constituent biological components of the vaccine, injection site reactions, age and sex. The aim of this study is to determine the incidence rate of adverse events following immunization with pentavalent vaccine (DTPw-Hep B-Hib (PRP-T) vaccine (pentavac) (adsorbed) is manufactured by Serum Institute of India ltd), which was introduced in Iran in November 2014. It is important to monitor vaccine-related adverse events because of the role of vaccine safety in immunization program success.

**Methods:**

This study was a mixed cohort study that included 1119 children less than 1 year of age. In 2015, the children were referred to Hamadan health centers to receive pentavalent vaccine at 2, 4 and 6 months of age. The data were collected from the parents of the children using a questionnaire that was administered either face-to-face or by telephone. The cumulative incidence of side effects and risk ratio was reported with 95% confidence intervals (CI). Chi-squared tests and logistic regressions were used to investigate the association between the variables.

**Results:**

The cumulative incidence rate of pentavalent-related adverse events during 48 h following immunization was estimated to be 15.8% for swelling, 10.9% for redness, 44.2% for pain, 12.6% for mild fever, 0.1% for high fever, 20.0% for drowsiness, 15.0% for loss of appetite, 32.9% for irritability, 4.6% for vomiting and 5.5% for persistent crying. There is no evidence for the occurrence of convulsion and encephalopathy among children who receive pentavalent vaccines.

**Conclusion:**

Further large studies with long time follow up are required to address rare events include convulsions, encephalopathy or persistent crying. However, Findings urge immunization programs to use pentavalent vaccinations and to continue implementing the current immunization program in children under 1 year of age.

## Background

Immunization is a fundamental component of public health policies for controlling infectious diseases. The vaccination program for smallpox effectively eradicated the disease from the world. Moreover, similar vaccination programs successfully led to the regional eradication of other infectious diseases, making vaccination one of the most reliable and cost-effective public health interventions [1]. According to estimations made by the World Health Organization (WHO), approximately two million deaths among children under 5 years of age can be prevented annually through the use of existing vaccines [2]. WHO has recommended the Expanded Program on Immunization (EPI), which integrates *Heamophilus influenza* type b (Hib) vaccine into the routine pediatric immunization program. Hib is a leading cause of life-threatening infectious diseases, including meningitis and pneumonia, that mostly affects children under 5 years of age [3, 4]. Hib is responsible for a significant proportion of the disease burdens of both developed and developing countries. Each year, Hib leads to approximately three million cases of serious illness and 400,000 to 700,000 deaths among children [5].

Some new vaccines, especially multivalent ones, have been added to the immunization program, primarily to add more antigens to vaccines. For example, in most countries, the tetravalent (DTP-Hib or DTP–HepB) or pentavalent (DTP-HepB-Hib) combination vaccines are currently being used instead of the trivalent vaccine (DTP) without the Hib or HepB component [6]. Combination vaccines for pediatric immunization schedules have contributed to a decrease in the number of clinic visits, logistical challenges, operational costs and injections and an increase in parental consent [7, 8]. Moreover, these vaccines improve individual adaptation to the virus and routine vaccination coverage. Therefore, the addition of antigens to existing vaccines with high coverage is considered an effective and appropriate strategy for protecting society from new diseases [9, 10].

Public trust in newly introduced vaccines can be strengthened by monitoring vaccine safety. Surveillance of adverse events following immunization will enable us to monitor the safety of immunization programs and thereby contribute to validating the immunization program. In this way, the undesirable adverse events of the immunization program can be effectively managed, and any inappropriate measures based on reports of adverse effect that may cause concern in society can be prevented [11–13]. As the pentavalent vaccine (DTPw-Hep B-Hib (PRP-T) vaccine (pentavac) (adsorbed) manufactured by Serum Institute of India ltd) has been part of the national pediatric immunization schedule of Iran since 2014, the study of adverse events associated with this vaccine seems to be of paramount importance. After assessing for reactogenicity following immunization with the pentavalent vaccine, we can take effective and useful action toward earning public trust and managing vaccine safety.

The aim of this study is to examine pentavalent vaccine safety and increase the amount of knowledge that might help policy makers in their decisions to continue introducing pentavalent vaccines to immunization programs.

## Methods

The sample of the present mixed cohort study was comprised of 1119 children under 1 year of age who were referred to Hamadan health centers to receive pentavalent vaccine at 2, 4 and 6 months of age. More specifically, a sample of approximately 370 children was examined at each point of the three scheduled doses of pentavalent vaccine. Only children under 1 year of age who received the first, second and third dose of pentavalent vaccine were eligible for inclusion in this study.

Urban health centers that are affiliated with the District Health Center in the city of Hamadan were selected using a randomized sampling method, and children under 1 year of age who were referred to these centers to receive pentavalent vaccine at 2, 4 and 6 months of age were recruited consecutively.

The data for the present study were collected through a self-constructed questionnaire from the parents of the children. Ethical approval for the present study was obtained from the Ethics Committee of the Hamadan University of Medical Sciences. Moreover, before the start of the study, parents of all the participants were informed of the study’s purpose and the procedure. We obtained verbal consent prior to administering the questionnaires. Because of using non-invasive approach at this study, we did not obtain written consent form. After completing the first part of the questionnaire, which included questions about the socio-demographic characteristics of the children and their parents along with some questions on their history of experiencing adverse events while receiving the last dose of pentavalent vaccine, we gave parents an information sheet that included questions related to the vaccination. After each vaccination (i.e. at 2, 4 and 6 months), they were asked to record any complications they observed in the children within the 48 h following the vaccination. After 48 h, we contacted parent by telephone to complete the second part of questionnaire. In total, we asked about the occurrence of 12 complications: (1) swelling, (2) redness, (3) pain, (4) mild fever, (5) high fever, (6) drowsiness, (7) anorexia, (8) restlessness, (9) vomiting, (10) long-term crying, (11) encephalopathy and (12) convulsion using standard definitions.

If parents reported a suspected occurrence of convulsion in the child, they were asked additional probing questions about all the symptoms they observed in the vaccinated child so that a specialist could make a definite conclusion concerning the occurrence of convulsion afterward. In a similar way, parents could report on other serious complications such as persistent crying and irritability. Parents were asked to report possible adverse events that they remembered in the case of a previous dose pentavalent/DTP vaccine. All children who received the first, second and third doses of the vaccine were followed up on by their parents (who were 98% mothers) 48 h after immunization to determine the incidence of complications.

The cumulative incidence of the outcomes studied (i.e. the adverse events after pentavalent immunization) were calculated with 95% confidence intervals (CI). Moreover, risk ratio (RR) was calculated by dividing the cumulative incidence of adverse events among male children to female children to explore gender differences. Chi-squared tests and logistic regressions were used to investigate the determinants of vaccine-related side effects. RR values have been approximated using logistic regression analysis. All statistical analyses were conducted at a significance level of 0.05 using SPSS software, version 20 (SPSS, Chicago, IL, USA).

## Results

In this study, of the children who received pentavalent vaccines in four health centers in Hamadan, 1119 children were included in the study for the final analyses, and all the pertinent data for these children were collected after they received three doses of pentavalent vaccine at 2, 4 and 6 months of age. The numbers of children at the first, second and third doses of vaccine were reported to be 373 (33.3%), 372 (33.2%) and 374 (33.4%), respectively. Moreover, 54.3% of vaccine recipients (*n* = 608) were male, whereas the remaining 45.7% (*n* = 511) were female. The present study demonstrated that the incidence rate of adverse events 48 h after pentavalent immunization in the children under study was estimated to be 15.8% (13.7–18.0) for swelling, 10.9% (9.2–12.9) for redness, 44.2% (41.2–47.2) for pain, 12.6% (10.7–14.6) for mild fever, 0.1% (0.0–0.4) for high fever, 20.0% (17.7–22.5) for drowsiness, 15.0% (12.9–17.2) for loss of appetite, 32.9% (30.2–35.8) for irritability, 4.6% (3.4–6.0) for vomiting and 5.5% (4.2–7.0) for persistent crying. However, there was no evidence for the occurrence of convulsion and encephalopathy among children who received vaccines. Table 1 presents the frequency of reactogenicity following immunization with pentavalent vaccine in the study group based on gender. The only significant gender differences were found with regard to persistent crying: the incidence of this complication in males was higher than in females (*p* = 0.01). We found no other significant gender differences in the incidence of other complications.

**Table 1:** The cumulative incidence rate of adverse events associated with pentavalent vaccination in male and female children and related risk ratios

Adverse events		Incidence (per 100 children)		*P*-value	Risk ratio (95% CI)^a^
		Sex			
		Male	Female		
Swelling	Yes	103(16.9)	74(14.5)	0.149	1.16 (0.88–1.50)
	No	505(83.1)	437(85.5)		
Redness	Yes	64(10.5)	59(11.5)	0.327	0.91 (0.65–1.27)
	No	544(89.5)	452(88.5)		
Pain	Yes	282(53.6)	213(41.7)	0.065	1.11 (0.97–1.27)
	No	326(53.6)	298(58.3)		
Mild fever	Yes	75(12.3)	66(12.9)	0.420	0.95 (0.70–1.30)
	No	533(87.7)	445(87.1)		
High fever	Yes	0	1(0.2)	0.457	NA
	No	608(100)	510(99.8)		
Drowsiness	Yes	123(20.2)	102(20)	0.486	1.01 (0.80–1.28)
	No	485(79.8)	409(80)		
Anorexia	Yes	91(15)	77(15.1)	0.514	0.99 (0.75–1.31)
	No	517(85)	434(84.9)		
Restlessness	Yes	204(33.6)	165(32.3)	0.351	1.03 (0.87–1.22)
	No	404(66.4)	346(67.7)		
Vomiting	Yes	29(4.8)	23(4.5)	0.474	1.05 (0.62–1.80)
	No	579(95.2)	488(95.5)		
Long-term crying	Yes	43(7.1)	19(3.7)	0.010	1.90 (1.13–3.22)
	No	565(92.9)	492(96.3)		
Encephalopathy	Yes	0	0	1	NA
	No	608(100)	511(100)		
Convulsion	Yes	0	0	1	NA
	No	608(100)	511(100)		
History of convulsion	Yes	3(0.49)	0	0.43	NA
	No	605(99.5)	511(100)		
Family history of convulsion	Yes	10(1.64)	10(1.9)	0.16	0.84 (0.35–2)
	No	598(98.3)	501(98.0)		

*NA* not applicable

^a^Female gender was considered as reference for RR calculation

With regard to the vaccine dose, the results of statistical analyses indicated that the frequency of adverse events due to pentavalent immunization in our sample were statistically significant for swelling (*p* = 0.002), pain (*p* = 0.002) and mild fever (*p* = 0.004). As shown in Table 2, more fine-grained analyses revealed that the frequency of swelling (*p* = 0.003), pain (*p* = 0.001), mild fever (*p* = 0.003) and redness (*p* = 0.02) were significantly higher after the first dose of vaccination compared to the second dose, but that children were more restless after the second dose than after the first (*p* < 0.001). Furthermore, the symptoms of mild fever were significantly higher in the third dose than in the second dose ((*p* < 0.001).

**Table 2:** Comparison of the cumulative incidence rates of complications in the first, second and third doses of the vaccination in children

Adverse events	Incidence (per 100 children) vaccine dose			*P*-value (1st and 2nd dose comparison)	*P*-value (2^nd^ and 3^rd^dose comparison)
	1st dose (2 months) *N* = 373	2^nd^dose (4 months) *N* = 372	3^rd^dose (6 months) *N* = 374		
Swelling	79(0.21)	50(0.13)	48(0.12)	0.003	0.068
Redness	50(0.13)	30(0.08)	43(0.11)	0.026	0.679
Pain	190(0.5)	143(0.38)	62(0.43)	0.001	0.162
Mild fever	45(0.12)	33(0.08)	63(0.16)	0.068	<0.001
High fever	1(0.002)	0(0)	0(0)	0.388	NA
Drowsiness	83(0.22)	72(0.19)	70(0.18)	0.310	0.725
Anorexia	65(0.17)	51(0.13)	52(0.13)	0.126	1
Restlessness	35(0.06)	115(0.3)	119(0.31)	<0.001	0.766
Vomiting	16(0.04)	19(0.05)	17(0.04)	0.510	0.510
Long-term crying	24(0.06)	16(0.04)	22(0.05)	0.210	0.510
Encephalopathy	0(0)	0(0)	0(0)	NA	NA
Convulsion	0(0)	0(0)	0(0)	NA	NA
History of convulsion	0(0)	1(0.002)	2(0.005)	0.387	0.488
Family history of convulsion	0(0)	14(0.037)	6(0.016)	<0.001	0.074

*NA* not applicable

In this study, we further examined the relationship between sex and adverse events following pentavalent vaccination by means of logistic regressions. As shown in Table 1, the results of the analyses indicated that there is no association between sex and adverse events following pentavalent vaccination. Furthermore, we examined the relationship between vaccination dose (i.e. first, second and third dose) and adverse events following pentavalent vaccination. As shown in Table 3, there is a statistically significant relationship between the vaccination dose and swelling (RR = 0.72 (95% CI: 0.59–0.89) and pain (RR = 0.85 (95% CI: 0.74–0. 99). These complications decrease with increasing age.

**Table 3:** Relationship between vaccination dose and the cumulative incidence rate of adverse events associated with pentavalent vaccine in children

Adverse events	Incidence (per 100 children)			RR^a^ (95% CI)
	First dose *N* = 373	Second dose *N* = 372	Third dose *N* = 374	
Swelling	79(44.6)	50(28.2)	48(27.1)	0.72(0.59–0.89)
Redness	50(40.7)	30(24.4)	43(35)	0.9(0.72–1.14)
Pain	190(38.4)	143(28.9)	162(32.7)	0.85(0.74–0.99)
Mild fever	45(31.9)	33(23.4)	63(44.7)	1.24(1.00–1.54)
High fever	1(100)	0(0)	0(0)	NA
Drowsiness	83(36.9)	72(32)	70(31.1)	0.89(0.74–1.07)
Anorexia	65(38.7)	51(30.4)	52(31)	0.87(0.71–1.06)
Restlessness	135(36.6)	115(31.2)	119(32.2)	0.9(0.77–1.05)
Vomiting	16(30.8)	19(36.5)	17(32.7)	1.02(0.73–1.44)
Long-term crying	24(38.7)	16(25.8)	22(35.5)	0.94(0.69–1.29)
Encephalopathy	0(0)	0(0)	0(0)	Not applicable
Convulsion	0(0)	0(0)	0(0)	Not applicable
History of convulsion	0(0)	1(0.2)	2(0.5)	3.45(0.5–23.74)
Family history of convulsion	0(0)	14(3.7)	6(1.6)	1.6(0.9–2.83)

*NA* not applicable

^a^Risk ratios values have been approximated using logistic regression analysis

## Discussion

The implementation of immunization is meant to protect individuals and society as a whole against vaccine-preventable diseases. Although advances in medicine have made vaccines reliably more effective with minimal adverse events, no vaccine can be found that is free from unwanted adverse events [11]. As the immunization schedule for Hib is similar to that of the DTP vaccine, it can be easily incorporated into the current immunization program [14]. There has been no report of incompatibility between the Hib vaccine and the DTP and hepatitis B vaccines. In general, each new combination vaccine containing the DTP vaccine is acceptable if there is no interference among multiple antigens. Physical and chemical interactions among the components of a combination vaccine and immunological interference may trigger undesirable changes in the immune response to vaccines [15]. Hence, in this study, we attempted to investigate the incidence rate of adverse events following immunization associated with pentavalent vaccine, which has been implemented as a combination vaccine in Iran for the first time. To the best of our knowledge, this is the first study that aims to provide evidence regarding the safety of pentavalent vaccine in Iran.

The results obtained in the present study showed that the most frequently reported reactogenicity associated with pentavalent vaccine was pain, with an incidence rate of 44.23%. This finding is in line with the results obtained by Edna in his meta-analysis, in which some minor reactions such as pain and redness were more prevalent among the children who received the combined vaccine [16]. The results of a study conducted in China revealed that the majority of the adverse events following immunization were reported to be non-serious events; fever and injection site reaction were the most common forms of reactogenicity experienced after immunization [17]. Moreover, in other Iranian studies on the adverse events associated with DTP and pentavalent vaccination, mild fever was found to be the most commonly experienced complication that occurred after vaccination [18–20]. Contrary to the above observation, in our study, the incidence rate of mild fever was a reported 12.2%. The main reason for the difference could be the use of acetaminophen prior to vaccination and a lack of sufficient parental attention to the child’s temperature. We observed no significant difference in the incidence rate of complications following vaccination between male and female children except for persistent crying, which was significantly more common among males than females.

We have cited some studies that assessing adverse events associated with the pentavalent vaccine with a separate formula or combinations. In a safety study among Indian infants [21], authors have monitored adverse events following a hexavalent vaccine during 1 month after immunization. They found that 37.9% infants experienced at least one injection site reaction. The corresponding value for systemic reaction was 54.6%. Wang YX et al. [22] in a clinical trial to evaluation the safety of a “combined Haemophilus influenzae type b-Neisseria meningitidis serogroup A and C-tetanus toxoid conjugate vaccine (Hib-MenAC)” found that there is no serious adverse events following immunization with Hib-MenAC. In a retrospective cohort study, Sadoh AE and his/her colleagues [23] have compared the prevalence of adverse events following pentavalent and DTP vaccines in Nigeria. They reported that the rate of pentavalent-related adverse reactions vaccine was higher than DTP one (22.2% vs. 13.5%). Similar studies in Iran have been implemented on different antigen [24].

A comparison of the complication incidence rates of the first and second doses of vaccination with pentavalent showed that all the adverse events were more frequently observed in the first dose of vaccination than the second, and this relationship was statistically significant. The only exception was restlessness, which had a higher incidence rate in the second dose of vaccination rather than the first. The meaningful reduction in the incidence rate of these complications with increasing age can be partly accounted for by several factors, including the increase of muscle tissue and deeper intramuscular injections. Moreover, mothers might be more sensitive and worried about the complications their children show after the first dose of vaccination and thus give more detailed reports of the complications their children experience after the first dose of vaccination than after subsequent doses. In addition, due to the experience they have gained after the first dose of the vaccination, some mothers may use acetaminophen prior to subsequent doses. The results of the statistical analyses showed that with the exception of mild fever, which was more common in the third dose of vaccination than the second one, there was no significant difference between the incidence rate of all other complications after the second and third doses.

### Study limitations

One limitation of the present study is that the limited sample size and the rarity of more serious complications such as convulsion, encephalopathy and persistent crying constrain the generalizability of the study results. Other limitations are the duration of follow up participants and lack of control group. It is evident that some of rare adverse events such as encephalopathy occur after 7–10 days following immunization or later. Therefore, similar studies with long duration follow up, larger sample sizes or the use of surveillance system data associated with adverse events after immunization are recommended. Consider to the above mentioned limitations, findings on mild and moderate reactions after pentavalent vaccine in this study as a field work in the new setting could support licensur studies.

## Conclusion

In general, the present findings on usual reactions should move immunization programs to use pentavalent vaccinations and continue implementing the current immunization program in children under 1 year of age. Moreover, the findings of this study show that giving some useful guidelines to parents with children under 1 year of age about avoidable adverse events following vaccination such as pain, redness and swelling is a necessary measure for effectively managing these complications.

## Abbreviations

EPI: Expanded Program on Immunization
Hib: Heamophilus influenza type b
WHO: World Health Organization
