# Correction to: Adverse events following immunization with pentavalent vaccine: experiences of newly introduced vaccine in Iran

**DOI:** 10.1186/s12865-017-0228-6

**Published:** 2017-10-05

**Authors:** Manoochehr Karami, Pegah Ameri, Jalal Bathaei, Zeinab Berangi, Tahereh Pashaei, Ali Zahiri, Seyed Mohsen Zahraei, Hussein Erfani, Koen Ponnet

**Affiliations:** 1 Social Determinants of Health Research Center, Hamadan University of Medical Sciences, Hamadan, Iran; 2 Department of Epidemiology, School of Public Health, Hamadan University of Medical Sciences, Hamadan, Iran; 3 Deputy for Health, Hamadan University of Medical Sciences, Hamadan, Iran; 4 Social Determinants of Health Research Center, Kurdistan University of Medical Sciences, Sanandaj, Iran; 5 Center for Communicable Diseases Control, Ministry of Health, Tehran, Iran; 6 Department of Communication Studies, Ghent University, Ghent, Belgium; 7 Faculty of Social Sciences, University of Antwerp, Antwerp, Belgium

## Erratum

After publication of the original article [1], the authors noticed that the percentages in Table 2 were incorrect.

**Table 2:** Comparison of the cumulative incidence rates of complications in the first, second and third doses of the vaccination in children

Adverse events	Incidence (per 100 children) Vaccine dose			*P*-value (1^st^ and 2^nd^ dose comparison)	*P*-value (2^nd^ and 3^rd^dose comparison)
	1^st^ dose (2 months) *N* = 373	2^nd^dose (4 months) *N* = 372	3^rd^dose (6 months) *N* = 374		
Swelling	79(21.2)	50(13.4)	48(12.8)	0.003	0.068
Redness	50(13.4)	30(8.1)	43(11.5)	0.026	0.679
Pain	190(50.9)	143(38.4)	62(16.6)	0.001	0.162
Mild fever	45(12.1)	33(8.9)	63(16.8)	0.068	<0.001
High fever	1(0.3)	0(0)	0(0)	0.388	NA
Drowsiness	83(22.3)	72(19.4)	70(18.7)	0.310	0.725
Anorexia	65(17.4)	51(13.7)	52(13.9)	0.126	1
Restlessness	35(9.4)	115(30.9)	119(31.8)	<0.001	0.766
Vomiting	16(4.3)	19(5.1)	17(4.5)	0.510	0.510
Long-term crying	24(6.4)	16(4.3)	22(5.9)	0.210	0.510
Encephalopathy	0(0)	0(0)	0(0)	NA	NA
Convulsion	0(0)	0(0)	0(0)	NA	NA
History of convulsion	0(0)	1(0.3)	2(0.5)	0.387	0.488
Family history of convulsion	0(0)	14(3.8)	6(1.6)	<0.001	0.074

*NA* not applicable

The correct version of Table 2 is published in this erratum.
